# Return to sport after conservative versus surgical treatment for pubalgia in athletes: a systematic review

**DOI:** 10.1186/s13018-022-03376-y

**Published:** 2022-11-11

**Authors:** Thiago Teixeira Serafim, Eliton Stanley Oliveira, Filippo Migliorini, Nicola Maffulli, Rodrigo Okubo

**Affiliations:** 1 Department of Physiotherapy, University of the State of Santa Catarina, Florianópolis, SC Brazil; 2 Department of Orthopaedic, Trauma, and Reconstructive Surgery, RWTH University Hospital, 52074 Aachen, Germany; 3 Department of Orthopaedic and Trauma Surgery, Eifelklinik St. Brigida, 52152 Simmerath, Germany; 4 Department of Medicine, Surgery and Dentistry, University of Salerno, 84081 Baronissi, Italy; 5 School of Pharmacy and Bioengineering, Faculty of Medicine, Keele University, Stoke-on-Trent, ST4 7QB England, UK; 6 Barts and the London School of Medicine and Dentistry, Centre for Sports and Exercise Medicine, Mile End Hospital, Queen Mary University of London, London, E1 4DG England, UK

**Keywords:** Groin pain, Physiotherapy, Pubalgia, Surgery, Treatment

## Abstract

**Background:**

To assess the time required to return to sport (RTS) after conservative versus surgical treatment in athletes for pubalgia.

**Methods:**

The PRISMA guidelines were followed. Pubmed, SportDiscus and Web of Science were last accessed on September 2022. All the studies investigating the time to RTS after conservative versus surgical treatment in athletes for pubalgia.

**Results:**

In total, 33 studies were selected for full text assessment, and 10 studies were included in the qualitative analysis. Seven studies reported data on conservative management, two on surgical management and one compared both. A total of 468 subjects were included for analysis. 58.7% (275 of 468) were soccer players, 5.9% (28 of 468) runners, and 3.8% (18 of 468) hockey players. Two studies did not specify the type of sport. The quality of the studies detailing the results of conservative management was higher than surgical procedures.

**Conclusion:**

This review highlights that individuals undergoing surgery for pubalgia may return to sport earlier than those receiving conservative treatment. However, conservative management should be considered before surgical treatment is indicated.

## Introduction

Pubalgia is common, especially in sports such as football, hockey, rugby, running, and tennis athletes[1]. Pubalgia is prevalent in males and in soccer players [2, 3]. Pubalgia manifests with pain in the inguinal region, impairing athletic performance [3]. Pubalgia can be due to acute trauma or to chronic overuse [4, 5]. Given the multifactorial aetiogenesis, the approach to manage pubalgia is challenging [6]. The management of pubalgia involves physiotherapy programs, pharmacological treatments, and surgical intervention [7].

Pubalgia negatively impacts athlete performance, causing prolonged absence or even prematurely retirement from sports [8]. Its treatment is a challenge, and finding the best way for the athlete to return to the sport is fundamental to his career or quality of life. For this purpose, this systematic review wished to assess whether there is a difference in the time required to RTS after conservative or surgical treatment using clinical trials. We also examined the various modalities of treatment, and the criteria used to clear the patients for RTS.

## Materials and methods

### Study protocol

This systematic review followed the Preferred Reporting Items for Systematic Reviews and Meta-Analyses (PRISMA) guidelines [9]. This study was registered in PROSPERO (ID CRD42018098922).

### Search strategy

The literature search was performed independently by two authors (TTS and ESO). The PICO algorithm was preliminary pointed out:

- P (Problem): pubalgia;
- I (Intervention): conservative management;
- C (Comparison): surgical management;
- O (Outcomes): return to sport time

The literature search was performed in Pubmed, SportDiscus, and Web of Science in September 12, 2022. The Medical Subject Headings (MeSH) terms were used: "Pubalgia" or "Groin pain" or "Osteitis pubis" combined with "Treatment" or "Physical therapy" or "Surgery" (Table 1). There was no time limit set for the search.

**Table 1:** Search strategy on electronic database

Databases	Terms	Results
Pubmed:		
#1	“Groin pain”	2057
#2	Pubalgia	158
#3	“Osteitis pubis”	389
#4	1 OR 2 OR 3	2423
#5	Treatment	12,478,128
#6	“Physical therapy”	98,927
#7	Surgery	5,309,247
#8	5 OR 6 OR 7	13,834,865
#9	4 AND 8	1980
SportDiscus:		
#1	“Groin pain”	688
#2	Pubalgia	104
#3	“Osteitis pubis”	140
#4	1 OR 2 OR 3	839
#5	Treatment	132,733
#6	“Physical therapy”	51,514
#7	Surgery	83,325
#8	5 OR 6 OR 7	224,796
#9	4 AND 8	523
Web of Science:		
#1	“Groin pain”	2123
#2	Pubalgia	254
#3	“Osteitis pubis”	453
#4	1 OR 2 OR 3	2429
#5	Treatment	5,794,057
#6	“Physical therapy”	43,090
#7	Surgery	3,637,162
#8	5 OR 6 OR 7	8,589,213
#9	4 AND 8	1761

All the titles of the resulting articles were screened by the author in a separate fashion, and, if of interest, the full-text was accessed. The bibliographies of the full-text were also accessed. Disagreements between the authors were solved by a third author (RO).

### Eligibility

All the published clinical studies which investigated the role of conservative and/or operative management for pubalgia in athletes were accessed. Comments, reviews, case reports, editorials, letters to the editor, technical notes were not eligible. Given the authors language capabilities, articles in English, Portuguese, and Spanish were considered. Only studies that reported quantitative data with regards to the RTS were included in the present investigation. Only studies that clearly indicated the nature of the treatment were eligible.

### Data extraction and outcomes of interest

Data extraction was performed by two reviewers (TTS and ESO). Patient demographic was extracted. Furthermore, study objective, type of treatment (surgical or conservative), characteristics of the intervention postoperative, duration of preoperative symptoms, return to sport time, return to sport rate, return to sport criteria and other results.

### Methodological quality assessment

To evaluate the methodological quality assessment, the Downs and Black checklist [10] was used. This checklist is composed by 27 items divided into 4 main categories: Reporting, External validity, Interval validity—Bias and Confounding, and Power. The final classification of the studies is based on the sum of each items: excellent (24–28 points), good (19–23 points), fair (14–18 points) and poor (< 14 points).

### Quality of evidence

To assess the quality of evidence, we used the Grading of Recommendations Assessment, Development, and Evaluation (GRADE). For the purpose of this review, we examined the following GRADE aspects: risk of bias, inconsistency, indirectness, imprecision. Based on these criteria, the quality of evidence of a study is classified as high, moderate, low, or very low [11].

## Results

### Literature search

**Fig. 1:**
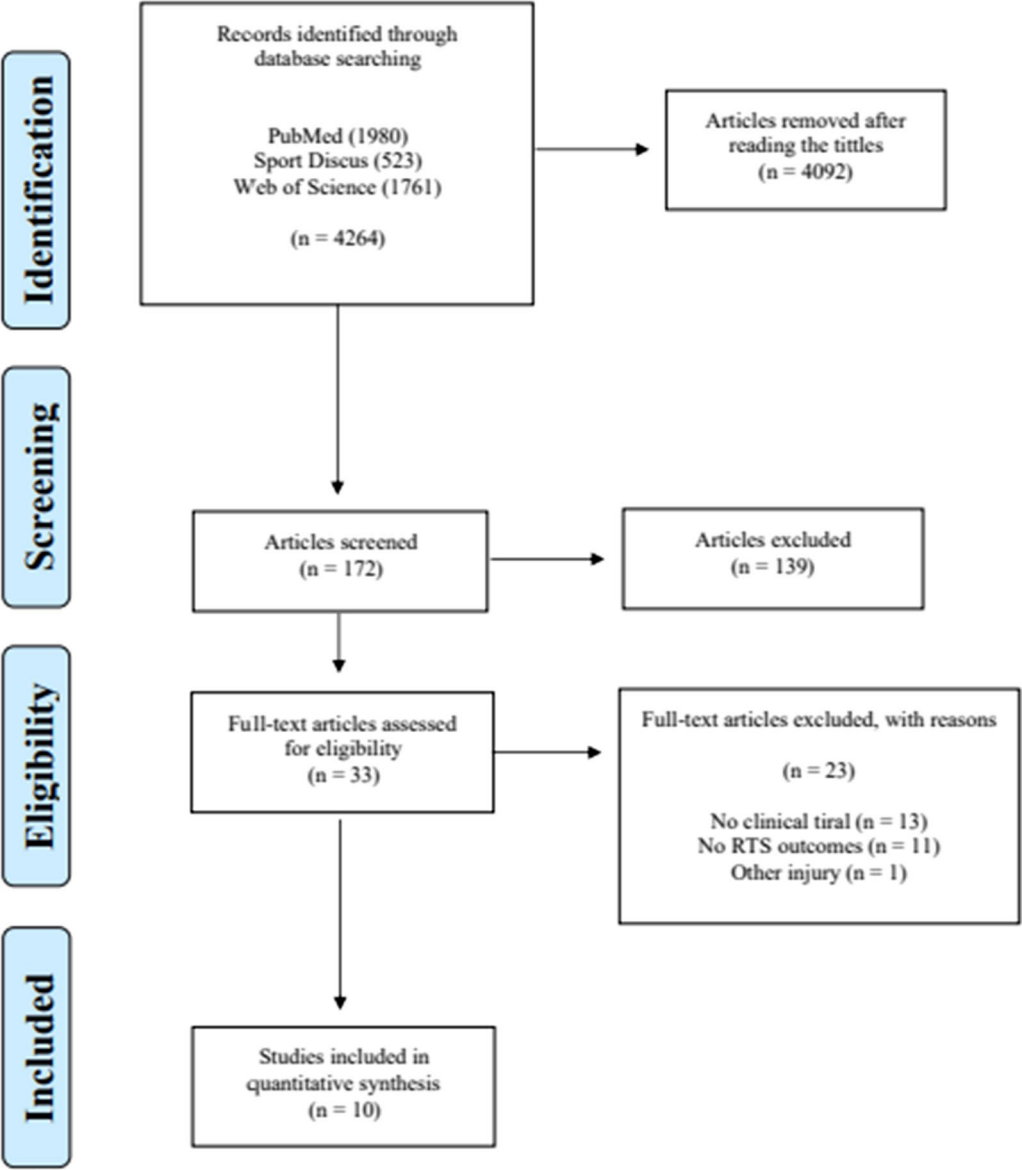
PRISMA flowchart of the literature search

The initial search identified 4264 articles. After reading the titles, 172 studies remained for reading the abstracts. After reading the resulting abstracts, other 139 articles were excluded as the title did not match the topic and not reported any quantitative data on RTS. Thus, 33 studies were selected for full text assessment, and 10 studies were included in the qualitative analysis (Fig. 1).

### Quality of studies

Applying Downs and Black checklist, two studies were classified as "Excellent" [12, 13], six were rated as “Good” [14–19], none were rated as “Fair”, and two studies [20, 21] were rated as “Poor”. The final value was 19.5 ± 5.0, attesting to this review a good quality of the methodological assessment (Table 2).

**Table 2:** Evaluation of the quality of studies with Downs and Black checklist

Study	Reporting	External validity	Internal validity	Power	Total	Classification
Harr et al. [20]	3	3	2	0	8	Poor
Holmich et al. [14]	6	3	10	1	20	Good
Weir et al. [16]	9	3	7	1	20	Good
Yousefzadeh et al. [17]	8	2	10	0	20	Good
Schoberl et al. [15]	7	3	10	1	21	Good
Yousefzadeh et al. [19]	8	2	10	0	21	Good
Gore et al. [18]	10	2	9	1	22	Good
Paajanen et al. [13]	9	3	11	1	25	Excellent
Sheen et al. [12]	10	3	11	1	25	Excellent
Mazbouh et al. [21]	6	2	3	0	11	Poor

### Quality of evidence

Based on the GRADE assessment (Table 3), five included analyses were classified as high quality [12, 13, 15, 16, 18], three were moderate quality [14, 17, 19] and two studies very low quality [20, 21].

**Table 3:** GRADE

Study	ECR	Risk of bias	Inconsistency	Indirectness (PICO)	Imprecision	Publication bias	Dose–response gradient	Confusion	Quality
Harr et al. [20]	Yes	SR	SR	SR	SR	Detectable	No	No	Very low
Holmich et al. [14]	Yes	SR	SR	No SR	No SR	Undetectable	No	No	Moderate
Weir et al. [16]	Yes	No SR	No SR	No SR	SR	Undetectable	No	No	High
Yousefzadeh et al. [17]	Yes	No SR	No SR	No SR	SR	Detectable	No	No	Moderate
Schoberl et al. [15]	Yes	No SR	No SR	No SR	No SR	Undetectable	No	No	High
Yousefzadeh et al. [19]	Yes	SR	No SR	No SR	No SR	Undetectable	No	No	Moderate
Gore et al. [18]	Yes	No SR	SR	No SR	No SR	Undetectable	No	No	High
Paajanen et al. [13]	Yes	No SR	No SR	No SR	No SR	Undetectable	Yes	No	High
Sheen et al. [12]	Yes	No SR	No SR	No SR	No SR	Undetectable	Yes	No	High
Mazbouh et al. [21]	Yes	SR	No SR	SR	SR	Undetectable	No	No	Very low

### Characteristics of the studies

Seven studies performed patient randomisation clinical trials. Seven studies reported data on conservative management, two on surgical management and one compared A total of 468 subjects were included for analysis. 58.7% (275 of 468) were soccer players, 5.9% (28 of 468) runners, and 3.8% (18 of 468) hockey players. Two studies [18, 19] did not specify the type of sport. Study characteristics is shown in greater detail in Table 4.

**Table 4:** Results of studies performing conservative strategies

Reference	Objective	Treatment	Return to sport time	Return to sport rate	Other results	Return to sport criteria
Holmich et al. [14]	To compare an active training (AT) programme with a conventional physiotherapy (PT) programme in the treatment of severe and incapacitating adductor-related groin pain in athletes	AT: 90 min, 3 × a week instructed by a physiotherapist. The active training was divided in 2 modules, made with strength and coordination exercises PT: 90 min, 2 × a week by one physiotherapist. The PT programme was made with stretching, contract-relax technique, laser, transverse friction massage and transcutaneous electrical nerve stimulation (TENS)	Mean time of 18.5 weeks (13–26 weeks)	AT group: 79% (23/29) subjects returned to sports PT group: 14% (4/30) subjects returned to sports	There was a significant linear trend towards better effect of the AT treatment Abduction ROM increased significantly in both treatment groups without difference between then Adduction strength improved significantly in the AT group compared with the PT group	Treatment was stopped when neither the treatment nor the jogging caused any pain The subjects and the physiotherapist decided when to stop the treatment
Weir et al. [16]	To compare the new therapy (Multimodal treatment) to the current therapy with the highest level of evidence, for the treatment of long-standing adductor-related groin pain, in a single blinded prospective randomized clinical trial	ETG: Six weeks focus in the exercises for strength and balance. After, phases of the return progressive to sport, witch slow jogging, straight sprints and cutting MMTG: This group utilizes a paraffin pack to warm the adductor muscle and stretch, finish with manual therapy at the warmed place	ETG: mean time of 17.3 weeks MMTG: mean time of 12.8 weeks	ETG: 55% (12/22) returned to sports MMTG: 50% (13/26) returned to sports	The VAS pain scores at 0 and 16 weeks during sports improved significantly in both groups	–
Yousefzadeh et al. [17]	Check the effect of the modified version of the Hölmich et al. protocol for the treatment of long-standing adductor-related groin pain (LSAGP) and aimed to evaluate its effects on athletes with this type of injury	The treatment occurred 10–12 weeks, 120–150 min, 3 × a week. Divided in 2 phases, compounded by strength, coordination and balance exercises, utilizing sliding boards, medicine balls, proprioceptive discs and soccer balls	Mean time of 12.06 weeks	86% (13/15) of subjects returned to sports	There was significant improvement in VAS pain scores for the legs adduction, THT and ESST Meaningful improvements were also found in the T-Test agility scores Hip abduction and adduction ROM and internal rotation increased significantly at the end of the treatment	Not established, but functional tests (HHD, THT, ESST and T-Test) are used
Schöber et al. [15]	To show the positive effect of a standardised treatment programme for symptomatic pubic overload in athletes with groin pain and osteitis pubis in a prospective randomized controlled study	Standardised nonsurgical treatment consists in a 3 phase program, with manual therapy, mobilization exercise, aerobic exercises, stretching, gradual exposure to sport movements, proprioceptive exercises, and strength exercises Group 1: shock wave therapy additionally to standardised treatment Group 2: there were utilized sham shock wave therapy additionally to standardised treatment Control group: stopped participation in sports activity	Group 1 (Shock wave therapy: mean time of 73.2 days (10 weeks approximately) Group 2 (Sham shock wave therapy): mean time of 102.6 days Control group: mean time of 240 days	95% (42/44) of subjects, of both groups, returned to sports	The VAS, Oswestry low back pain and HOOS showed fewer complaints already 1 month after the beginning of therapy (p < 0.001 for both groups) Subjects of the control group frequently experienced recurrent groin pain during the first year after the beginning of therapy (26/51; 51%)	The return-to-sport decision was made by the subjects, the team coach and the physician
Yousefzadeh et al. [19]	To objectively evaluate the effect of Holmich protocol-based exercise therapy on LSAGP	The treatment occurred 10–12 weeks, 90-120 min, 3 × a week. Divided in 2 phases, compounded by strength, coordination and balance exercises, utilizing sliding boards, medicine balls, wobble board and soccer balls	Mean time of 14.2 weeks	78%% (11/14) of subjects returned to sports	There was significant improvement in VAS pain scores for the squeeze test, THT, ESST, between hip internal rotation before and after treatment in the affected limbs was significant Meaningful improvements were also found in the T-Test agility scores, for THT and ESST functionality	Not established, but functional tests (HHD, THT, ESST and T-Test) are used
Gore et al. [18]	The aim of this study was to determine if anterior groin pain (AGP) affects kinematics and kinetics during hurdle hop task using a continuous waveform analysis approach and if so, how these affected kinematics and kinetics compare to uninjured controls following return to sport	The exercise program was divided on 3 levels, 4 × per week alternating between, inter-segmental control, strength and running drills, without supervision (physiotherapist assessed each patient’s progress at regular intervals)	Mean time of 9.14 weeks	100% (65/65) of subjects returned to sports	There was significant improvement in maximum adductor squeeze score (at 0°, 45° and 90°) HAGOS scores improved in 5 out 6 subscales, seven of eighteen kinematic and kinetic variables were no longer significantly different between the two groups	HAGOS Adductor squeeze test HHT The authors suggest the kinematics and kinetics variables may represent the factors most related to return to sport
Paajanen et al. [13]	To compare the efficacy of video-assisted, preperitoneal insertion of polypropylene mesh to nonoperative treatment of suspected sportsman’s hernia in athletes, including magnetic resonance imaging (MRI) studies	Nonoperative treatment: 8 weeks,3 times a week for 90 min, exercise program supervised by a club physiotherapist The exercise program focus was strength, balance training and reduce pain (with TENS)	Full return to sports activity was achieved in 20% and 27% after 1 and 3 months, respectively At 12 months 15 (50%) subjects, had achieved full return to sports	1 Month Follow up: (20%) 3 months follow up: (27%) 12 months follow up: (50%)	Of the 60 study subjects, unilateral pain was found in 40 (67%) and bilateral pint in 20 (33%) Complete relief of pain, after 1 month, was achieved at 14 subjects only in operative group. None of subjects from nonoperative group has reached 0 on VAS Complete relief of pain, after 3 months, was achieved on 27 subjects at operative group, and 2 at nonoperative group Complete relief of pain, after 12 months, was achieved on 29 subjects at operative group, and 14 at nonoperative group	–
Mazbouh et al. [21]	To evaluate the effect of low intensity of exercise treatment based on MHP on long-standing ALrGP	The treatment consisted of MHP suggested by Yousefzadeh et al. 2018 under the supervision of a trained sport physiotherapist. Note that, the participant will continue his normal pain-free training with the team and he rest if pain is felt at any time during team training. The minimum duration of treatment was 10 weeks. Intensity identification session was held one week before the protocol treatment starts to exclude any effect of muscle fatigue and low exercise intensity was well defined. For applying low resistance in dynamic exercises, we identified the 1RM for each exercise in the pre-treatment session and we applied low intensity exercises at 40% of 1RM. The intensity can be changed to the targeted percentage of 1RM by using weights or elastic bands with different resistances as needed. Note that, 1RM will be assessed every two weeks as a progression of exercise intensity. For balance exercising, in addition to warm-up and stretching exercises, the intensity was not changed because it is considered as functional and overall exercising. In addition to Copenhagen Adduction exercises, where the intensity was identified based on previous studies	Mean time of 17.3 weeks	100% (10/10) of subjects returned to sports	–	Not established, but functional tests (Biodex, SEBT) are use, and Pain 2/10 (VAS)

*AGP* Anterior Groin Pain,* ALrGP* Adductor Longus related Groin Pain,* AT* Active training,* ESST* Edgren Side Step Test,* ETG* Exercise therapy group,* HAGOS* Copenhagen Hip and Groin outcome score,* HHD* Handheld dynamometer,* HHT* Hurdle hop test,* HOOS* Hip disability and osteoarthritis outcome score,* LSAGP* long-standing adductor-related groin pain,* MHP* Modified Hölmich Protocol,* MMTG* Multi-modal treatment program group,* MRI* Magnetic resonance imaging,* PT* Physiotherapy,* ROM* Range of Motion,* RTS* Return to sport,* TENS* Transcutaneous electrical nerve stimulation,* THT* Triple hop test,* VAS* Visual analogic scale

### Conservative management

A total of seven studies verified the effects of conservative treatment on athletes with pubalgia. The time to RTS ranged from 9.14 weeks [18] to 18.5 weeks [14], and the percentage of athletes who were able to RTS ranged from 14% [14] to 100% [18, 21] (Table 4). Four studies reported criteria for discharge. Data concerning the conservative management are shown in greater detail in Table 5.

**Table 5:** Results of studies performing surgical strategies

Reference	Objective	Surgery	Characteristics of the intervention postoperative	Duration of preoperative symptoms	Return to Sport Time	Return to sport rate	Other results	Return to sport criteria
Harr et al. [20]	To demonstrate a suture repair and a rectus and adductor longus tenotomy technique for sports hernias	Suture herniorrhaphy with adductor tenotomy	Exercise program: 2–6 week, focused on stretching, ROM, aerobics, core and weightlifting activities	–	6–8 weeks	100% (22/22) subjects returned to sports	A total of nine (40.91%) subjects received prior steroid injections, but all continued to have persistent symptoms requiring surgery	–
Sheen et al. [12]	The aim of this study was to compare the effectiveness of Open minimal suture repair (OMR) and TEP repair for the treatment of sportsman’s hernia	OMR Group TEP Group	Conventional non-steroid anti-inflammatory drugs and/or paracetamol were prescribed for postoperative pain relief. Patients we allowed to walk and lift up 20 kg 2 Days after surgery: running and cycling were allowed 1 week: free training	Over 6 weeks	At 2 weeks, 1 subject from OMR and 4 from TEP group, were cleared to full return to sport At 1 month, 16 subjects from OMR and 18 from TEP group, were cleared to full return to sport At 3 months, 25 subjects from OMR and 31 from TEP group, were cleared to full return to sport At 12 months, 28 subjects from OMR and 32 from TEP group, were cleared to full return to sport. Only 2 subjects from both were at partial level of return to sport and only 1 do not return (from OMR group)	OMR group: 90% (28/31) subjects returned to sports TEP group: 94% (32/34) subjects returned to sports	There was a decrease between preoperative scores and those measured at 4 weeks in both groups. Relief of pain (VAS < 20 points) during sports activity 4 weeks after surgery was achieved in 14 of 31 subjects in the OMR group and 24 of 34 in the TEP group	–
Paajanen et al. [13]	To compare the efficacy of video-assisted, preperitoneal insertion of polypropylene mesh to nonoperative treatment of suspected sportsman’s hernia in athletes, including MRI studies	TEP, and for 6 patients who presents simultaneous insertion tendonitis of the adductor magnus or longus, and open tenotomy was performed after the TEP surgery under the same anaesthesia	Training with full activity was initiated when the pain allowed	3–6 months	Full return to sports activity was achieved in 67% and 90% after 1 and 3 months, respectively At 12 months, 29 (96%) subjects, had achieved full return to sports	1 Month Follow up: (67%) 3 months follow up: (90%) 12 months follow up: (97%)	Unilateral pain was found in 40 (67%) subjects and bilateral pint in 20 (33%) subjects Complete relief of pain, after 1 month, was achieved at 14 subjects only in operative group. None of subjects from nonoperative group has reached 0 on VAS Complete relief of pain, after 3 months, was achieved on 27 subjects at operative group, and 2 at nonoperative group Complete relief of pain, after 12 months, was achieved on 29 subjects at operative group, and 14 at nonoperative group	–

*MRI* Magnetic resonance imaging,* OMR* Open minimal suture repair,* ROM* Range of motion,* RTS* Return to Sport,* TAPP* laparoscopic transabdominal preperitoneal,* TENS* Transcutaneous electrical nerve stimulation,* TEP* Endoscopic total extraperitoneal,* VAS* Visual analogic scale

### Surgical management

Three studies described the results of surgical treatment for pubalgia [12, 13, 20]. Time to RTS ranged from 6 weeks [20] to 12 weeks [12, 13]. The postoperative rehabilitation protocols were well structured in two studies [12, 20] and ranged from immediate return to sport [12] to 6 weeks [20]. The studies which investigated the actual rate of RTS reported a success rate of 90% [12, 13] to 100% [20]. Data concerning the surgical management are shown in greater detail in Table 6.

## Discussion

### Time of return to sport

The most clinically relevant finding of this study was that athletes who underwent surgery for the treatment of pubalgia started to RTS three weeks after the index procedure. However, clinical trials reporting the outcome of surgery are lacking. In general, the studies detailing the results of conservative management show that these athletes RTS three weeks later than those managed surgically. However, the investigations on conservative management are more articulated and include more and better validated outcome measures.

The most common form of surgery is Total Endoscopic Extraperitoneal (TEP) repair of a sportsman hernia [12, 13]. In the studies where this procedure was performed, athletes returned to the sports on a median time of 12 weeks. Other surgical procedures resulted in RTS at six [20] to 12 weeks [12].

The time from onset of symptoms to surgery ranged from six weeks [12] to 6 months [13]. The fact that patients with a short duration of symptoms underwent surgery may have been a factor contributing to their rapid RTS, with an average of 4.3 weeks. These athletes probably underwent less invasive or less extensive surgery [22, 23]. In any case, it is recommended that conservative treatment should be attempted before surgery is recommended, although it is unclear for how long conservative management should be implemented [7, 24, 25].

### Rate of return to sport

The rate of RTS in athletes who underwent surgery was 90% [12, 13] to 100% [20, 21], while it ranged from 14% [14] to 100% [18] in those who underwent conservative management, showing great differences in rates of RTS between the different conservative management regimens.

Holmich et al. [14] divided their participants into two groups: their novel physical exercises activity group vs conventional physiotherapy group, in both groups undertaken for 12 weeks. The conventional physiotherapy group received only passive techniques, as in Weir et al.’s [16] investigation. Gore et al. [18] used an active treatment with a more structured time-dependent program.

Other studies demonstrated how important exercise therapy is to increase the RTS rating. Ramazzina et al. [6] showed that active treatment provides a faster RTS. Abouelnaga et al. [26] demonstrated that active rehabilitation training resulted in a higher rate of RTS and reduced the pain associated with a sports hernia.

### Other results

Explicit criteria to allow an individual to RTS were described in five studies [14, 15, 17–19], all of them reporting the results of conservative management. Except for the two studies by Yousefzadeh et al. [17, 19], all used different criteria for RTS.

Functional tests [27], such as hop tests [28] and Star excursion balance test [29], should be part of the assessment process. Only three studies used functional tests [17–19]. However, normative values are unclear, and athletes may perform well and still have symptoms [30]. It is possible that the functional tests in this field do not engage the relevant muscles involved in pubalgia.

The addition of clinical tests should be performed to monitor athlete readiness to RTS. The absence of pain in the tests such as Copenhagen five-second squeeze [31], FADIR test, FABER test, abdominal test and absence of palpation pain [32] should, for example, be considered. Only two studies used a clinical test (the squeeze test). Gore et al. [18] tested the athletes at three angles (0º, 45º and 90º) of hip flexion and compared the values obtained before and after treatment. Yousefzadeh et al. [19] also used the squeeze test but did not specify angulation.

Athletes with pubalgia often demonstrated reduced mobility [33] and strength in the hip [34]. When allowed to RTS, athletes should have a difference in range of motion of the hip of less than 5 degrees [34]. Muscle strength differences should not exceed 10% to 15% [33], and the ratio between agonist/antagonist contraction should be above 80%. A ratio between adductor and abductor muscles below 80% is associated with a 17-fold increase in adductor injury [24].

Only one in one study was a patient reported outcome measure standardized questionnaires used [18], and the Copenhagen Hip and Outcome Score (HAGOS) [31] could be introduced in routine clinical practice.

The sport contributing most athletes with pubalgia was soccer, followed by running and ice hockey. All these sports involve unipodal support [35], associated in some with sudden change of direction, and excessive use of repetitive ballistic movements such as kicking and hopping [36].

### Studies quality and evidence level

Two studies scored “Excellent” [12, 13] in Downs and Black evaluation [10], and six were classified as “Good” [14–19]. The main issue was internal validation [10]. Most of the studies were randomized clinical trials, but more quantity and quality studies, especially on operative treatment [12, 13, 20, 21], are needed.

More studies were classified in high quality of evidence [12, 13, 15, 18, 33] in GRADE analysis [11], meeting the results found in the Downs and Black evaluation. The main difficulty found in the studies was in the item regarding the dose–response gradient. However, as the analysis was made by clinical trials, the quality of the evidence has a greater tendency to be high.

### Practical implications

Return to sport after treatment of athletic pubalgia should involve a multifaceted assessment process. Obviously, neither approach (operative or conservative) can ensure that a given athlete will return to sports. Conservative treatment is classically recommended before surgery is performed. However, the length of conservative management before failure of such modality is declared is undefined.

Even if successful, conservative management of pubalgia resulted in slower return to sport compared with operative treatment, but it should consider that the studies are not directly comparable in terms of criteria of inclusion of athletes, and outcome measures. If conservative management, surgery should be considered, as it allows a relatively fast return to sport, provided that a well-controlled and active postoperative rehabilitation regime is introduced.

### Limitations

Some points are important to be considered in this systematic review. The different methods used between the studies make it difficult to generalize the results.

The description of the diagnosis of pubalgia was not always clear in all studies, with different ways of diagnosing it. Consequently, the different treatments used, whether surgical or conservative, influence the non-standardization of outcomes. Regarding the outcomes, the different health indicators used and the fact that few have criteria for return to sport makes the heterogeneity between the studies even greater. This fact also contributed to the failure to carry out a meta-analysis.

Follow-up studies can be more reliable to assess the success of return to sport, a fact that did not always occur and also occurred in different periods between the studies. Postoperative rehabilitation needs to be better described in surgical studies, as it is also part of treatment success.

Studies with better methodological controls, including some with a larger sample, are important to take such results to a larger population, adopting greater external validity.

## Conclusion

This review highlights that individuals undergoing surgery for pubalgia may return to sport earlier than those receiving conservative treatment. However, conservative management should be considered before surgical treatment is indicated. If surgery is undertaken, an active rehabilitation program should be preferred. Active rehabilitation programs should be the stalwart of conservative treatment. The quality of the studies detailing the results of conservative management was higher than surgical procedures. For future studies, it is important to use standard measures and criteria for return to sport.

## Data Availability

All data generated or analysed during this study are included in this published article.

## Abbreviation

RTS: Return to sport
